# An IoT-Based Wristband for Automatic People Tracking, Contact Tracing and Geofencing for COVID-19

**DOI:** 10.3390/s22249902

**Published:** 2022-12-16

**Authors:** Sharanya Mahapatra, Vishali Kannan, Srinidhi Seshadri, Visvanathan Ravi, S. Sofana Reka

**Affiliations:** 1 School of Electronics Engineering, Vellore Institute of Technology, Chennai 600127, India; 2 Centre for Smart Grid Technologies, Vellore Institute of Technology, Chennai 600127, India

**Keywords:** contact tracing, geofencing, radio frequency identification, coronavirus disease, global positioning system, data analysis

## Abstract

The coronavirus disease (COVID-19) pandemic has triggered a huge transformation in the use of existing technologies. Many innovations have been made in the field of contact tracing and tracking. However, studies have shown that there is no holistic system that integrates the overall process from data collection to the proper analysis of the data and actions corresponding to the results. It is critical to identify any contact with infected people and to ensure that they do not interact with others. In this research, we propose an IoT-based system that provides automatic tracking and contact tracing of people using radio frequency identification (RFID) and a global positioning system (GPS)-enabled wristband. Additionally, the proposed system defines virtual boundaries for individuals using geofencing technology to effectively monitor and keep track of infected people. Furthermore, the developed system offers robust and modular data collection, authentication through a fingerprint scanner, and real-time database management, and it communicates the health status of the individuals to appropriate authorities. The validation results prove that the proposed system identifies infected people and curbs the spread of the virus inside organizations and workplaces.

## 1. Introduction

The outbreak of the coronavirus disease (COVID-19) has had a huge impact on technologies related to healthcare [1]. Worldwide lockdowns, closure of business organizations and educational institutions, hospitals overwhelmed with patients, and government and healthcare professionals implementing various rules and regulations to bring the spread of COVID-19 under control have become common. The drawbacks of the facilities and services provided by various healthcare organizations worldwide were brought into the limelight because of COVID-19. Healthcare workers (HCWs) have faced many challenges during the pandemic [2]. Contact tracing is a major challenge faced by healthcare professionals. Following the concept that prevention is better than a cure, terminating the spread of the virus before it takes a toll on us is essential. It is vital to identify any contact with infected people and to ensure that they do not interact with others. The rapid community spread of COVID-19 is likely to increase if authorities are unable to successfully isolate patients and guarantee that contacts can separate themselves from others, necessitating severe mitigation efforts to contain the virus. Contact tracing involves contacting an infected/suspected infected person, collecting data from the person, and quarantining them. In this case, contact tracing depends on the data provided by the person, which are unreliable and time-consuming to collect. Contact tracing teams have encountered adversity, such as incorrect data, a lack of public responsibility and solidarity, a lack of knowledge, fear, stigmatization, and attacks and harassment.

Multiple coronavirus variants have been detected and are currently under investigation [3]. Each new variety raises new problems, such as whether people are more likely to become ill or whether COVID-19 vaccines are still effective [4]. As the virus evolves through mutations, new variants of concern emerge, such as the delta (B.1.671.2) variant, which is approximately twice as infectious as the earlier Omicron (B.1.1.5291) and BA lineages. The COVID XE variety is a hybrid of two Omicron variant strains, BA.1, the parent strain, and BA.2, which is a more contagious strain [5]. Since January 2020, the World Health Organization (WHO) has been evaluating and investigating the progression of severe acute respiratory syndrome coronavirus 2 (SARS-CoV-2) in partnership with many nations, expert alliances, ministries, organizations, and scientists. The identification of specific variants of interest (VOIs) and variants of concern (VOCs) in late 2020 to address global tracking and studies, and ultimately to notify the ongoing response to the COVID-19 pandemic, has urged the classification of specific VOIs and VOCs. In many countries where VOCs are widely diffused, public health and social methods, such as infection prevention and control, have proven to be effective in reducing COVID-19 cases, hospitalizations, and fatalities.

Most business organizations and educational institutions are resuming in full swing [6,7]. Therefore, maintaining safety guidelines and safeguarding communities against the spread of coronavirus has become a major concern. Organizations should keep track of people’s health status and travel details so that they can be used whenever necessary. This process should be automated and cost-effective for all organizations to maximize utilization. Healthcare technology has grown tremendously in recent years. Every day, new inventions and products are designed for the advancement of humankind. All of these innovations should be made available to everyone in a more feasible manner. In the early days of the pandemic, state and district governments used several models to conduct contact tracing. However, the stigmatization associated with testing positive for COVID-19, as well as the imposition strategy used by the authorities to combat the disease, such as lockdowns and the distinction of confinement zones, struggled to motivate self-assessment and reporting by ordinary citizens. Currently, hospitals in India and other countries still face a lack of technology for the prevention of new cases, which exacerbates the lack of manpower. The present Internet of Things technology in our hospitals might be helpful for personal health monitoring [8,9,10] but does not pay much attention to contact tracing, contact identification, and follow-up [11].

The use of radiofrequency identification and a global positioning system in IoT-based tracking has proven to be one of the solutions that help prevent the spread of COVID-19 and minimize its effect [12]. Automating the contact tracing process, keeping track of people’s health status, maintaining the travel history of a person, and maintaining a secure environment by geofencing the location helps both the authorities and the people of the organization with increased efficiency and reduced time to maintain healthy circumstances [13,14,15,16,17]. Therefore, the main motivation of this study is to maintain safety guidelines in an organization, thereby maintaining a safe and secure environment for people through prevention rather than cure. The proposed system helps achieve the goal of a robust territory within an organization more economically and efficiently, which is essential for a developing nation such as India.

The key contributions of this study are summarized as follows.

- We developed an RFID and GPS-enabled wristband to monitor, track, and trace the travel path of the user.
- We developed an IoT system that collects data from the RFID and GPS-enabled wristband and uploads data to the cloud, analyzes them, and alerts authorities based on the results.
- We developed a fingerprint-based scanning system to provide additional authentication and verification of the user.
- We developed a geofencing algorithm to create virtual boundaries around buildings, premises, or blocks and monitor the movement of infected people.
- We created a robust and modular database that allows maintaining a record of the details, health status, and location of the users within the campus/organization.

## 2. Related Work and Contributions

We reviewed the existing work in the field of contact tracing and systems that ensure adherence to COVID-19 safety guidelines. Table 1 summarizes the related work and its outlook measures. In light of new information and data on international deployments in digital contact tracing technology, Ref. [18] provides a complete overview of solutions relevant to digital contact tracing in terms of their techniques and technologies. The authors proposed a prototype based on digital contact tracing using IoT while considering open difficulties, such as scalability, privacy, and flexibility, as well as promising future research avenues. It also discusses some of the challenges that this technology may face, such as low smartphone penetration, ethical issues about privacy, and limitations in the transparency of data.

Ref. [19] describes an automated tracing and tracking system based on the Internet of Things that uses the most cost-effective RFID tags and individual smartphones as readers to detect potential interactions. High-frequency passive RFID tags have been proposed, which operate within a range of one meter and over a frequency of 13.56 Megahertz (MHz) and extract energy from electromagnetic induction from the reader. It involves tracing and tracking persons who have been close to suspected individuals to take further measures such as quarantine and treatment. However, the paper only presents a partial idea of the data stored in the database and does not offer robust information, as proposed in our work.

Ref. [20] describes a set of rules and procedures based on the Internet of Things paradigm that enables the connectivity of various devices to monitor and track a patient’s vital parameters and alert the connected healthcare organization to potentially life-threatening situations. These parameters include gathering health vitals, air quality monitoring, and location detection. However, in this study, it was necessary to identify and differentiate between missing data and repeated measurements. The system proposed in this study follows a sequential flow of data, which eliminates the need for segregation and identification of correct data. All collected data were labeled and ready for analysis.

Using COVID-19 clusters, Ref. [21] presents a unique intelligent contact tracking and cluster prediction technique. To aid in COVID-19 analysis, the solution contains a phone app linked to a wearable device, as well as unique intelligent IoT characteristics such as sophisticated data processing and smart data visualization incorporated inside the system. While existing contact tracing techniques are useful, the proposed tool is likely to be the only one that can conduct both contact tracing and COVID-19 prediction. However, this depends on users having an app on their phones linked to the wearable device. User adaptability and installation of another app become liabilities, which we have attempted to overcome in our proposed work.

A low-cost and lightweight IoT node, a fog-based machine learning tool for data analysis and diagnosis, and a mobile phone application were demonstrated in Ref. [22]. This IoT-enabled node continuously monitors health indicators such as body temperature, rate of respiration, and blood oxygen saturation and then updates the information in the mobile app to show the user’s current health state. However, the prevention of disease is of utmost importance to assuage the pandemic, and simply monitoring patients would not achieve that. In our proposed work, we address this issue by focusing on contact tracing, contact identification, and follow-up.

Ref. [23] presented a study of Signature Home, a self-operating IoT-based geofencing algorithm that uses waterproof Bluetooth low-energy wristbands that are uniquely paired with smartphones belonging to patients and uses environmental network facility identifiers such as Wi-Fi access points and cellular networks as the home signature to monitor patients cost-effectively. This paper defines a geofencing approach that is dependent on a secondary device such as a mobile phone. In this case, the wearable band pairs with the mobile device through Bluetooth. If the band is not in proximity to the phone, it is assumed that the user is away from the geofenced area. Ease of use is not guaranteed, as wearable and mobile apps must be properly calibrated by following a set of steps.

Ref. [24] proposed a system to trace both home contacts and business contacts by using RFID to fetch the details of the near contacts stored in the cloud database (AWS) and by designing an Android application to track the location of contact details through GPS. However, in the contact-tracing process, only the coordinates at which the reader detects a tag are recorded. Therefore, contact tracing depends on a set of discontinuous GPS coordinates at random locations. Our work continuously records and stores both the scanned location coordinates of users and the live GPS location coordinates of users in an online repository. Thus, we obtained an accurate path that could be visualized in detail for contact tracing.

Bluetooth Low Energy (BLE) is another technology available for contact tracing or human monitoring. Several BLE research works [25,26,27,28,29] are proposed in the literature to address the issues such as contact tracing, people tracking, occupancy detection and monitoring in office spaces, social interactions, etc. Ref. [30] proposed a smart contact tracing application that uses the mobile phone’s BLE signals and machine learning techniques to identify infectious individuals. IoT-based occupant tracing applications were also proposed [31,32,33] to address occupant tracing in the building or indoor environments and occupant-based actuation in smart commercial buildings. However, the BLE has some concerns. It has a low level of security and can be hacked at any time. Additionally, the BLE has a small bandwidth. Under certain circumstances, BLE can lose its connection, and it is not recommended for sending large volumes of data.

Ref. [34] proposed a situation-aware method to identify early symptoms of COVID-19 and alert people about the condition to consider taking precautionary steps. This system acquires data from the sensors present in a smartwatch, analyzes them, and generates an alert signal to the user based on the situation.

Ref. [35] presents a review of Internet of Medical Things (IoMT) applications in the healthcare domain. It discusses technology, adoption across the globe, suitable devices, interfacing protocols, security issues, research gaps, and implementation challenges.

**Table 1:** Summary of related work.

Paper	Design Methods	Limitations and Strengths	Outlook Measure
[18]	This paper contrasts the centralized and decentralized architecture of digital contact tracing employing IoT.	A scalable and automated system for contact tracing; digital contact tracking enhances the process of contact tracing.	To increase the voluntary utilization of such technologies, the confidence of the users in the preservation of their privacy-sensitive information in the current digital contact tracking applications can be bolstered.
[19]	High-frequency passive RFID tags that function within a range of one meter and over a frequency of 13.56 Megahertz (MHz) and draw energy from the electromagnetic induction from the reader.	The proposed work primarily contributes to efficiently and economically tracing and tracking the persons who have been near the suspected individuals to further take the required measures such as undergoing quarantine and treatments.	Future work that incorporates the analysis of the risk of such persons affected by the infection will be analyzed based on the scoring pattern, which is based on the parameters such as a person’s age, sex, comorbid factors, and many other factors.
[20]	Collection of health vitals Monitoring air quality Detection of location Setting and processing of rules using IoT frameworks, tools, and technology.	Monitor live statistics; alerts issued when readings cross the threshold; readings stored in the database; lower burdens on hospital staff.	By incorporating more intuitive alerts, the dashboard can be improved. It would be great to be able to see a patient’s average statistics regularly. When the real system runs, it is also crucial to figure out how to tell the difference between missing data and repeated measurements.
[21]	Comprehensive analysis of various IoT-based applications for contact tracing and patient monitoring, along with the challenges imposed by such devices.	Existing contact tracing tools are beneficial. The suggested tool is probably the only one that can perform both contact tracing and prediction of COVID-19. Addressing concerns and issues about security and privacy.	Cyber-attacks pose a security and privacy risk to IoT devices due to insufficient authorization and verification, weak web interface protection, or a lack of encryption. If we are to deal with patient health data, we must solve these issues.
[22]	Wearable IoT device Smartphone app A decision-making system—the fuzzy inference system Proximity detection using BLE and NFC Server and networking using Wi-Fi standard protocol Representational state transfer (REST) API and IPv4 or IPv6	Observing physical (or social) distance is one strategy to slow the transmission of viruses until a vaccine becomes available. Contagious diseases have a lower likelihood of spread by developing improved surveillance, healthcare, and transportation networks.	Contact tracing apps have been deployed in several nations. These apps, on the flip side, track only a patient’s history and location, and alert users if any person contracts COVID-19 in the locations the user has recently frequented. As a result, we must devise cost-effective and technologically optimized contact tracing and social distancing processes.
[23]	Waterproof BLE wristbands which are low-cost Smartphones Geofencing algorithm	This system checks whether the user is located within a geofenced perimeter. This system preserves privacy, is responsive and efficient, adapts to changes in diversity easily, is highly accurate, and is economical.	Wi-Fi should be made available to everyone. Should be improved in such a way that it can be used in rural areas. The system should be made cost-effective. Correct information to be provided by the user. Data integrity should be taken into concern.
[24]	Temperature prediction Storing of data to AWS cloud server Trace contacts using RFID Trace persons using GPS Issuing of alert	Helps to collect patient details smartly; helps in predicting the contacted person list. Patient vitals are stored in the cloud; this helps in issuing alerts while approaching a person with a high temperature.	In future work, a further collection of data and analysis of the same can be included with the prototype.

## 3. Proposed System Design

In this section, the design approach is described. Our system is designed in such a way that helps guarantee that individuals adhere to public health rules, which will directly contribute to minimizing the rate at which the virus spreads across the population, thereby dampening the peak. With a slower spread, few patients require critical care for serious illnesses on any given day. The main concepts required to understand the operation of this system are RFID technology and geofencing.

The use of RFID technology in a wristband can help create a safe environment for users [36]. Each wristband has a unique ID and can be detected using a second device that may be handheld or freestanding. The second device can be found on the premises of an organization, such as schools and offices. A short scan of the wristband might instantly display a “cannot enter” or “enter” signal on the gadget, indicating an individual’s health state. Status codes or classifications, such as healthy, recovered, and quarantined, were used.

The operation of the wristband and database is under the authority of the organization. It is responsible for checking the status of people trying to enter the premises. The organization will also have each person’s location data, which will help it trace his/her path if he/she tests positive later. The organization also has the responsibility to alert other people inside the premises of the number of sick people they might have come in contact with. The organization is also responsible for informing others of the location points of a person who tests positive.

### 3.1. Methodology

Each person in an organization will be provided with an RFID wristband, which will have a unique ID, along with GPS tracking. The unique ID will be linked to the relevant details related to a particular person, such as name, live location, and health status, and will be stored in cloud systems, such as Google Cloud. Thus, RFID readers, who act as location checkpoints, are present. These RFID readers will be located in different areas inside the organization’s premises, such as at the entrance and blocks. When the fingerprint which is used for authentication and the RFID and GPS wristband is scanned, the person’s information, location coordinates, and health status are reflected in the database. The status of a person is observed and the person is permitted accordingly. Furthermore, there is a facility that can change the user’s health status. The organization is located in a geofenced location. If an unhealthy/sick person attempts to enter an organization’s premises, an alert is issued. Those who must be isolated are monitored continuously. If it is necessary to track a person’s whereabouts inside an organization continuously, then their routes can be traced using the GPS visualization tool. Periodically, an email regarding the daily records of health conditions inside the organization is sent to all its members. A website with restricted access was developed to control and access the database about the details and health conditions of the organization’s members. The proposed system assumes the wearing of a wristband is compulsory in an organization. So, it may add some discomfort to the users. Additionally, there may be electromagnetic exposure associated with the RFID tag. However, the level of radiation is considerably low in intensity and short in duration. Furthermore, the application works better when there is a real-time integration with government applications, such as Aarogya setu, to fetch the health status of the members of the organization.

### 3.2. Wristband

**Figure A1:**
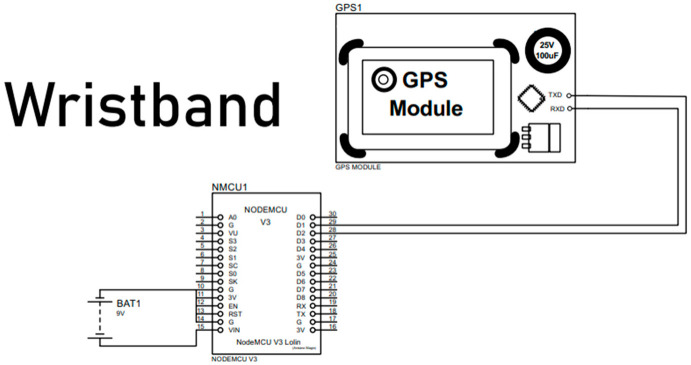
Circuit diagram of GPS and NodeMCU connection in the wristband.

The wristband side of our prototype consists of an RFID wristband that contains essential user information and the user status that would be used to validate a user’s entry inside the organization’s premise. In addition, the prototype included a GPS module that continuously monitored and tracked the location coordinates of the user. The GPS module used for creating the prototype was the NEO-6M GPS, which was programmed using NodeMCU. The circuit diagram of the wristband components is shown in Figure A1 in Appendix A.

### 3.3. Reader

**Figure A2:**
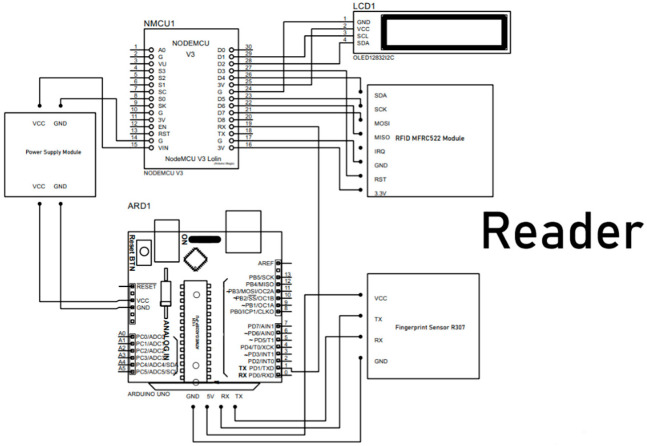
Circuit diagram of the RFID reader.

The reader consists of an RFID reader, a fingerprint module, and an LCD. The circuit diagram of the RFID reader is shown in Figure A2 in Appendix A. The RFID reader used for this purpose operates at a frequency of 13.56 MHz and is connected to a NodeMCU. The fingerprint module used for creating the prototype is the R307 fingerprint scanner, which is connected to an Arduino that aids in processing information received by the fingerprint scanner.

### 3.4. User Interface

A robust web application aids in protecting user data and ensuring highly limited access, that is, only to officials within an organization who are going to be in charge of this process. In addition, while handling large databases and multiple user entries, a website makes data visualization more convenient than a mobile app. Furthermore, websites can be opened on all devices (mobile, desktops, and laptops). In addition, bringing new updates or applications across all mobile devices through a website is much easier. Finally, because we did not extract any information from the user’s phone, a website serves the basic requirement conveniently and cost-effectively.

### 3.5. IoT System Design Architecture

**Figure 1:**
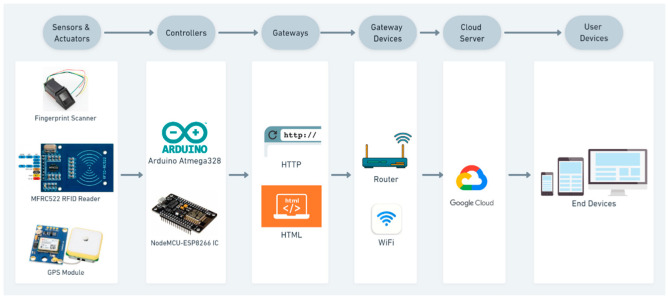
IoT system architecture of the proposed system.

Figure 1 depicts the IoT system architecture used in this study: The sensors responsible for the input of data are the MFRC522 RFID reader, fingerprint scanner, and Neo 6M GPS module. The ESP8266 IC controls the data flow from the sensors to the database, whereas the Atmega controller of Arduino controls the data coming from the fingerprint sensor. The gateways used were HTTP and HTML, the data were sent over HTTP to Google spreadsheets from the NodeMCU, and live location visualization was performed using HTML. The Wi-Fi module of the NodeMCU sends data to the cloud over the Internet. These data can then be visualized on end devices such as PCs and mobile phones.

### 3.6. Data Flow Architecture

**Figure 2:**
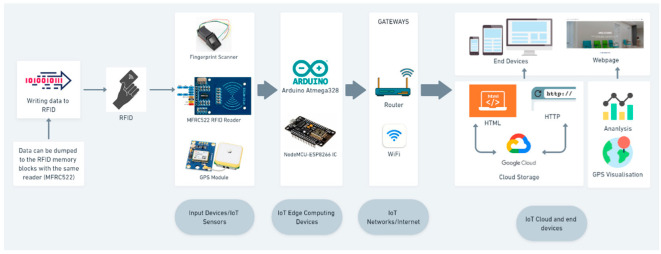
Data flow architecture of the proposed system.

Figure 2 explains the sequence of the data flow. The data can be dumped into RFID memory blocks by using the same reader (MFRC522). The passive 13.56 MHz RFID cards that we used had a memory of 1 kb. The 1 Kb EEPROM’s memory is divided into 16 sectors, each with four blocks, for a total of 64 blocks. Each block is composed of 16 bytes. After the data have been written to the RFID memory blocks, they can be read by the reader with a single scan.

The data from the RFID reader and GPS modules flow through the NodeMCU, whereas the serial data from the fingerprint sensor flow through the Arduino microcontroller. The user data are then sent to Google Cloud by calling the HTTP port, and the live GPS data are visualized using HTML. The data are stored in Google spreadsheets, and data analysis can be visualized on the end devices. We also created an interactive website that takes the data from the spreadsheets and makes them available to the authorities for final analysis and visualization.

## 4. Implementation of Proposed System

This section describes the implementation of the proposed system, the flow diagrams, and the algorithms. Our proposed system involves the sequential occurrence of events and data flows. To obtain this process, RFID and GPS enable wristbands to be issued by an organization. All students and employees were given one device for each. The default status in every RFID wristband is “HEALTHY”. As the users get tested for COVID-19 and receive their test results, if the results are negative, the status remains “HEALTHY”. If the user tests positive for coronavirus, the hospital, which previously collected his/her institutional details during the test, will share the user’s status with his/her organization. The organization then updates the new status of the user’s wristband.

**Figure 3:**
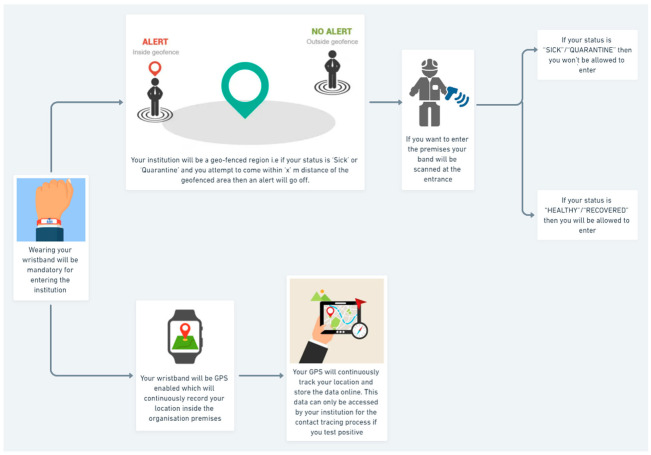
Flow diagram of COVID-19 contact tracing process.

The organization will refer to the GPS and RFID reader data and inform other people who might have come in contact with the infected person. At the end of the day, an email will be sent to all the users, alerting them about the number of sick/quarantined people who were on the premises, so that others can get themselves tested. The entire process continues in a loop. Thus, from Figure 3, we can see that the operation of the wristband and the database is under the authority of the organization. They are responsible for checking the status of people trying to enter the premises. The organization also has the responsibility to alert other people inside the premises of the number of sick people they might have come in contact with.

**Figure 4:**
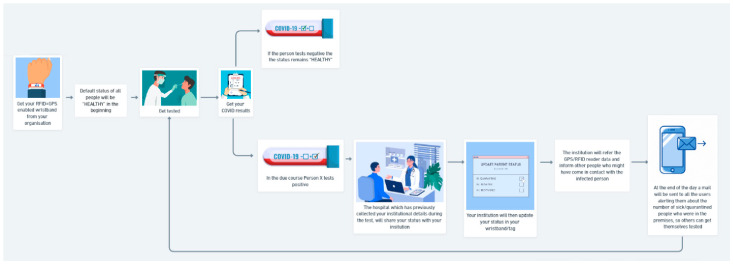
Overall block diagram of automatic people tracking and contact tracing system.

Contact tracing flow is the most vital part of this research. Figure 3 illustrates a contact tracing flow diagram that explains the technical aspects that are working in the background of the process flow. While Figure 4 describes the entire process of the proposed system. Here, we assume that the organization has made the wearing of wristbands compulsory. Thus, wearing it is mandatory for all users before entering the organization’s premises. The organization will be a geofenced region, i.e., if the user’s status is “SICK” or “QUARANTINE” and he/she attempts to come within “x” m distance of the geofenced area, then an alert will go off. The geofencing process is explained in detail in the following section. If the user wants to enter the premises, their band is scanned at the entrance by the RFID reader. If the user’s status is “SICK”/“QUARANTINE”, then he/she will not be allowed to enter. If the status is “HEALTHY”/“RECOVERED”, they are allowed to enter. All wristbands are GPS-enabled and continuously record the user’s location inside the organization’s premises. The GPS continuously tracks the user’s location and stores the data online. These data can only be accessed by the institution in the contact tracing process if any user tests positive.

### 4.1. Code Level Flow

**Figure 5:**
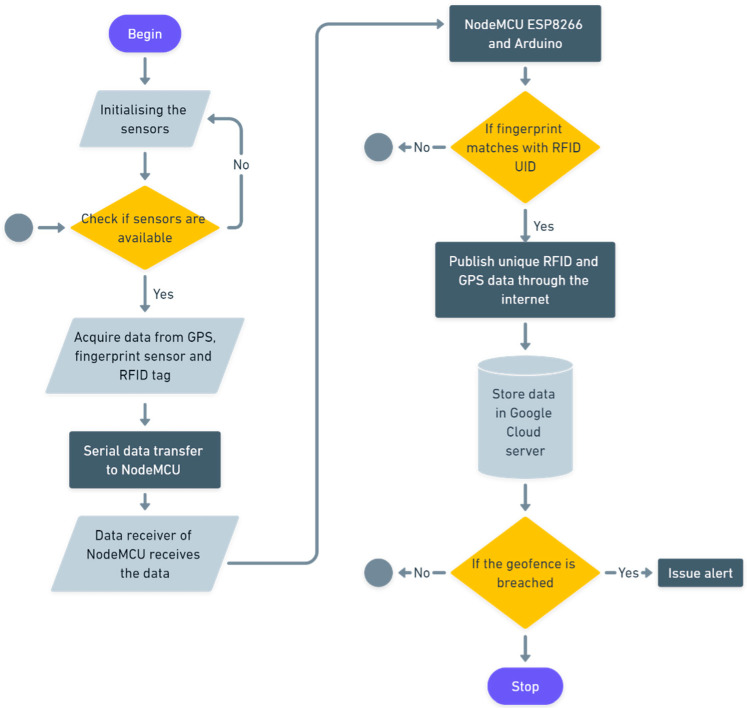
Flow chart of data collection, verification, and storage.

The flow diagram shown in Figure 5 depicts the operation of the code that controls RFID and GPS data. This algorithm, based on the Arduino Integrated Development Environment (IDE) platform, explains in detail the operation of the code.

### 4.2. Geofencing

**Figure 6:**
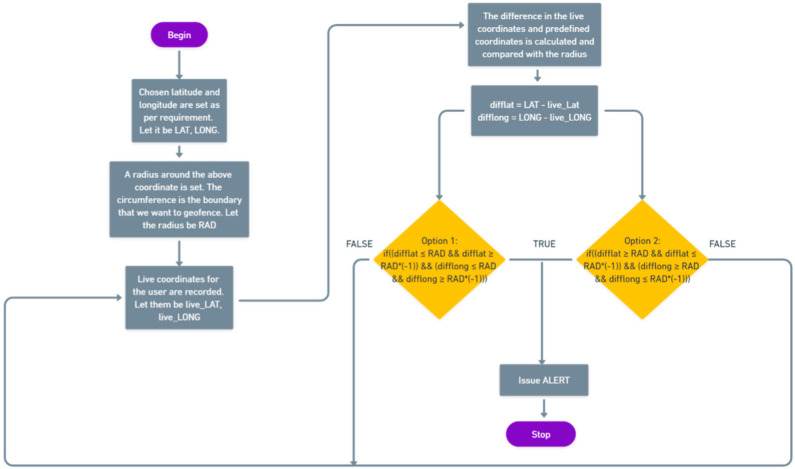
Flow chart for geofencing logic of the system.

Geofencing is a location-based service in which a mobile device or RFID wristband enters or exits a virtual boundary set up around a geographical area, known as a geofence, and an app or other software uses GPS, RFID, Wi-Fi, or cellular data to activate a pre-programmed action. Depending on how a geofence is set up, it can send push notifications, SMS, email, etc., allowing us to track how users travel around the premises. In this study, specific blocks inside the campus/premises were geofenced if required. The geofence can work in two ways: (a) issuing an alert if a person goes out of the geofenced area and (b) issuing an alert if a person tries to enter the geofenced area. Both options can be chosen according to the requirements. Administrators can use geofencing to set up triggers such as push notifications and email alerts. This can also be used to increase user awareness. The logic behind geofencing is illustrated in the flow diagram shown in Figure 6. This algorithm, based on the Arduino IDE platform, explains the operation of the code behind geofencing in detail.

### 4.3. Data Analysis and Visualization

We intend to protect user data and ensure highly limited access, that is, only to officials within an organization who are going to be in charge of this process. When handling big databases and multiple user entries, a website makes data visualization more convenient than a mobile app. As we do not extract any information from the user’s phone, a website serves the basic requirement more conveniently and cost-effectively.

We created a website that holds all information and actions required to operate the entire system. The website has the following features. (a) We can enter the website for the first time by clicking on the “Enter” button on the lightbox that pops up. (b) The website homepage, which is open to all, can be viewed. The homepage contains data on services provided, opening hours, and contact information. (c) However, a person viewing a website cannot access any database for data analysis without logging in. For extra security, only those authorized to access the website will be able to log in. They have to provide their email ID, and after we approve the information, the authorities are allowed to log in. (d) After logging in, the “Update Status” and “User Details” pages can be accessed. (e) On the “Update Status” page, all information, both overall and corresponding to each user, is available. (f) The data files, daily data analysis, GPS visualization, and the “Update user status” button will be available. (g) The “Update user status” is connected to the “User Details” page, where after entering the necessary information about the user, his/her health status can be changed according to his/her test reports. (h) There is also an option for operators to go to their profiles and make any changes to their information if required.

## 5. Results and Discussion

**Figure 7:**
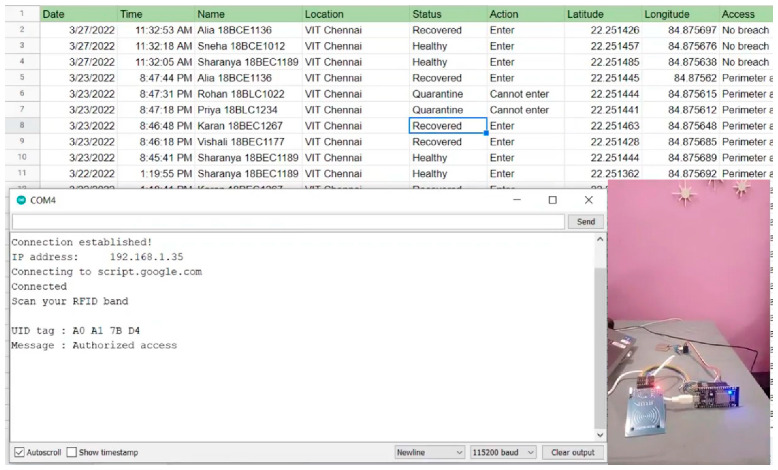
Experimental setup along with a status update in the COM port window and log screen.

**Figure 8:**
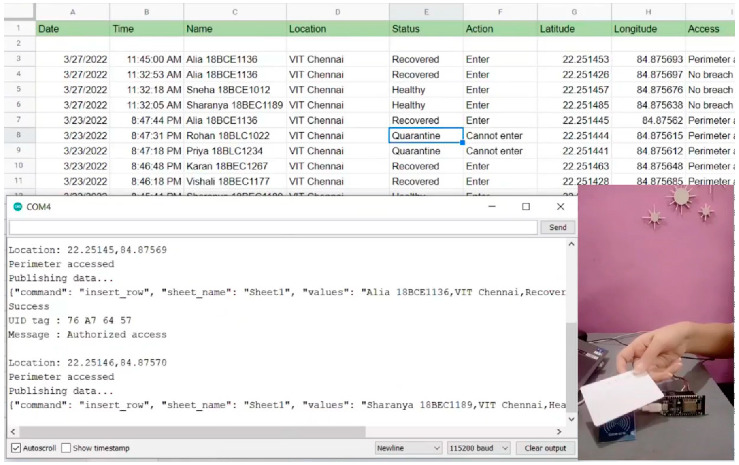
Location and status update in the COM port window and log screen after the RFID tag was scanned.

The experimental set-up of the proposed system hardware, along with the COM port window of Arduino IDE and the respective log screen is shown in Figure 7. The COM window shows the scanning and publishing of user information and the log screen display the live user information. Figure 8 illustrates the scanning of RFID, publishing of respective tag ID, location, and health status of the subject in the COM as well as log window. Each person in an organization will be provided with an RFID tag, which will have a unique ID along with GPS tracking. The unique ID will be linked to the user details such as name, live location, and health status. Fingerprint verification helps to authenticate the users. The fingerprint of the user is mapped with the respective RFID tag to prevent any misuse of identity. A geofencing algorithm was developed to create virtual boundaries for the virus-infected user. It defines the longitude, latitude, and radius of each infected user and keeps monitoring them. The algorithm issues an alert message whenever the user moves out of the geofenced area or any other user enters the geofenced region. Table 2 highlights the novelty of our proposed work in contrast to the existing works.

### 5.1. Log Datasheet

**Figure A3:**
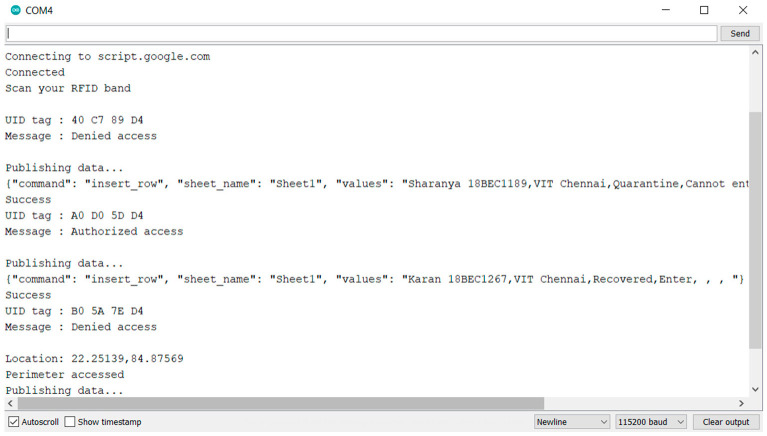
The COM window of Arduino IDE showing the scanning and publishing of user information.

**Figure A4:**
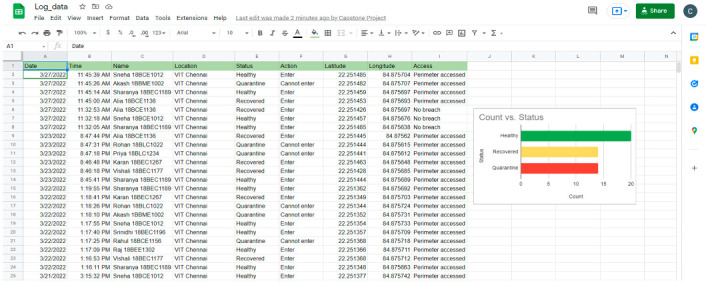
Log data page with live user information.

The log datasheet is used to collect the data whenever the RFID wristband is scanned. Figure A4 in Appendix A presents the typical log data page with live user information. In the COM window, the “Scan RFID band” message is displayed. When the wristband is scanned in the COM window, the unique ID, message, location, and whether the perimeter is accessed or not breached are displayed, and the data are published in the log datasheet. Figure A3 in Appendix A presents the COM window of Arduino IDE, showing the scanning and publishing of user information.

The wristband was scanned and the data were simultaneously published on the sheet. A successful message is displayed in the COM window. The first column in the sheet is the date and time of the card being scanned. Then, the following information is obtained: the name of the person, location in an organization, the different places where the wristband has been scanned, the status of the person (recovered, quarantine, healthy), action (whether the person can or cannot enter), latitude, longitude, and access to the organization. Access is granted according to geographical location and status. When a person enters a geofenced area, the message “Perimeter accessed” is published to the datasheet, and when the person is quite far from the area, a “No breach” message is published. When the person’s status is quarantine, the action is “Cannot enter”; accordingly, when the person is healthy, the action is “Enter”. These changes can be noted in the datasheet. The count vs. status chart is updated automatically as the wristband is scanned, and the number of healthy, recovered, and quarantined wristbands is counted and updated automatically.

**Figure 9:**
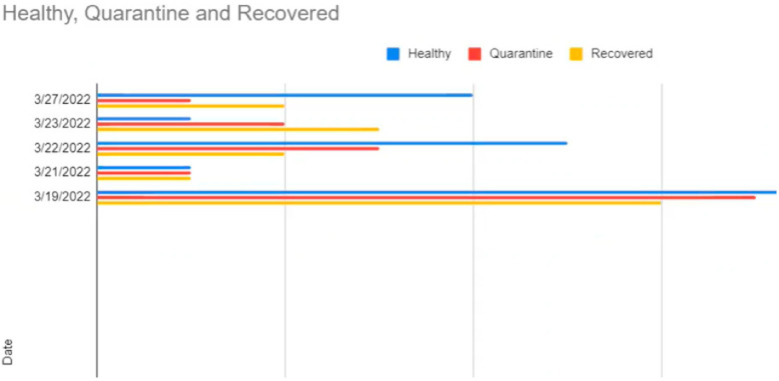
Information sheet for daily analysis of recorded data.

On sheet 2, the list of student names, their email IDs, the subject, the body part, and the count were also updated automatically using the formula. When in sheet 1, the wristband is scanned automatically, and the chart in sheet 1 and count in sheet 2 are updated. This information is primarily used to send emails. In sheet 3, the day-wise analysis, which is the entire history along with a date and the count of healthy, quarantined, and recovered individuals, can be visualized. This particular chart shown in Figure 9 was also published as a separate URL and sent along with the email.

### 5.2. GPS Datasheet

**Figure 10:**
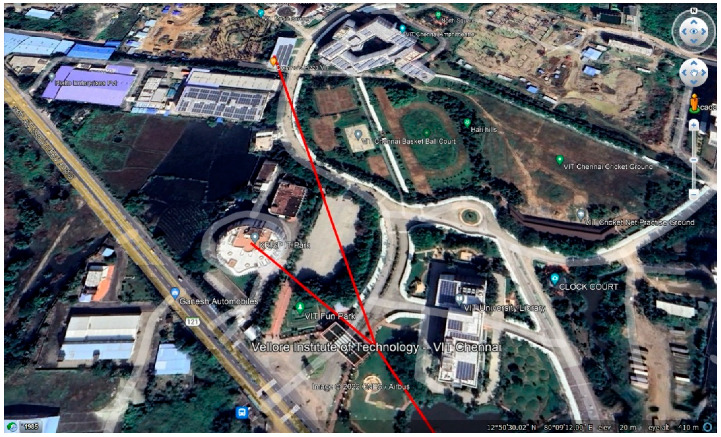
Detailed mapping of a user’s path for the contact tracing process.

**Figure A5:**
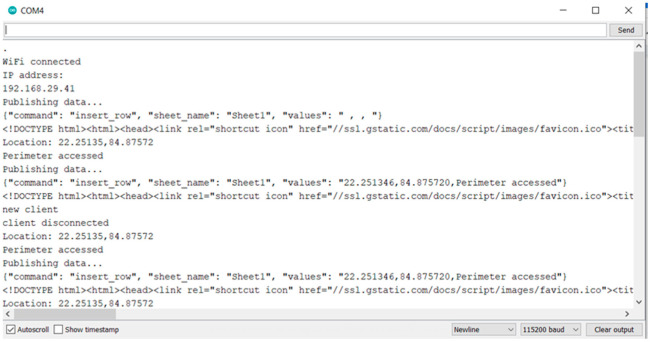
COM window of Arduino IDE showing the publishing of individual GPS data.

In the GPS datasheet, the continuous GPS data of a particular user are logged. The date, time, latitude, and longitude of one user were recorded and published on the sheet with a time interval of 6–7 s. Data were entered into the sheet when the GPS was turned on. In the COM window, the message is displayed when the data are being published, and a successful message is displayed when the data are written in the sheet. Figure A5 in Appendix A displays the COM window showing the individual GPS data. Google Maps were used to determine the current location of a person. When data are entered into the current location, the person can be visualized using the IP address. The red mark on Google Maps indicates the current location of the person. To see the entire route of the person, that is, the complete route of the person being continuously entered into the Excel sheet, the Excel sheet needs to be converted into a KML file, a keyhole markup language file, so that when the file is opened on Google Earth, the entire route traveled by the person is visualized. Figure 10 depicts the mapping of a user’s path for the contact tracing process. The red line on Google Earth shows the entire root traveled by the person.

### 5.3. Web Portal

A website that integrates all the tasks and functions of this study was created. The site “COVID-19 status monitoring” has a welcome page and a list of the services provided, the wristband, updating status, GPS monitoring, and a few other professional details such as contact ID and open hours.

On the website, there are provisions for changing the status of a person. The first and last names, user IDs, and health statuses should be updated. Once the changes have been made and the submit button has been clicked, the respective authorities receive an email. The status is updated at the backend, and changes can be observed in the log datasheet when the wristband is scanned again.

**Figure A6:**
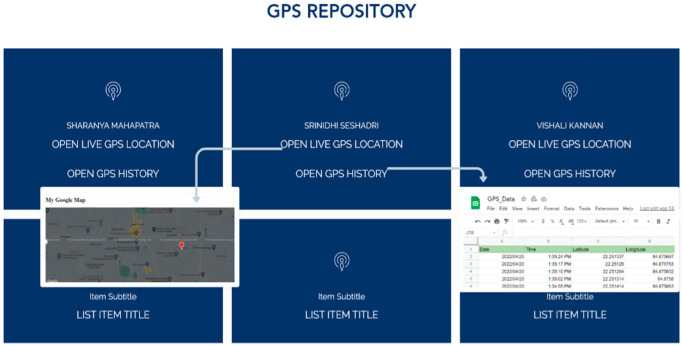
Respective links for GPS history and live location.

For “GPS visualization”, there is a provision to change the Excel sheet into a KML file that can be viewed on Google Earth. The website will also have a “GPS repository” for every person in the organization, as presented in Figure A6 in Appendix A. The live location where the wristband was scanned last will be opened by clicking the “open live GPS location button”, and the entire GPS history of the person will also be saved here and can be opened by clicking the “open GPS history” button.

### 5.4. Update Status

**Figure A7:**
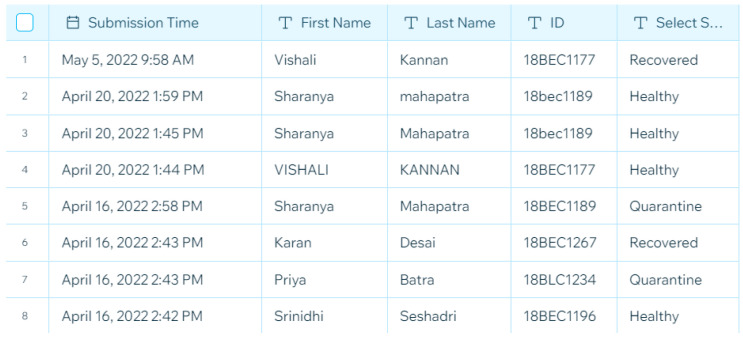
Overall status update track record.

Updating the status of users is an important aspect of this study. To update the status of the user, the “COVID-19 status monitoring website” can be used. Once the changes have been made and the submitted button has been clicked, the concerned authorities will receive an email with all details filled in. This change is also updated in the “Status Update” table, which can be viewed by the authorities. Figure A7 in Appendix A illustrates the sample status update record. These changes were noted and updated in the RFID wristband at the back end. When this card was scanned again, a new status was entered into the log datasheet. The database of the application and the web portal have restricted access. Login credentials are required to access the user information. Thus, the data collected through wristbands are accessible only to authorized people.

### 5.5. Sending Updates to the Users

Along with daily data collection and analysis, users were informed about the current status of their surroundings. We used Integromat software to send automated emails to users at periodic time intervals. The email ID was registered with the site, and an Excel sheet was created with all the required details added to the scenario. The contents of the Excel spreadsheet were updated automatically. The counts of the healthy, quarantined, and recovered are sent, and the day-wise analysis link is attached to the email. An email was sent from the registered email ID to the list of recipients added to an Excel spreadsheet. The email can be scheduled once a week, every day, or every month, according to the requirements of the organization. In this way, users are constantly updated about their organization’s status and can prepare themselves accordingly.

## 6. Conclusions

In this research work, we developed a fully automated IoT system to track people, trace contacts, and define virtual boundaries for COVID-19-infected people. The presented solution can be deployed in educational institutions or organizations to quickly identify COVID-19 patients and avoid the further spreading of the virus. The RFID and GPS-enabled wristband not only tracks the present location of the user but additionally, it records the entire travel path of the user and their contacts. The fingerprint scanner helps to authenticate people and avoids any misuse of wristbands. The proposed geofencing algorithm not only defines the boundaries for infected people but also checks whether the infected people stayed within the geofenced boundary. Furthermore, the algorithm generates an alarm whenever the infected people move out of the designated boundary. The proposed system integrates all the modules in real time and communicates the health status, travel path, and contact history to authorized people through email and a secure web portal.

### Limitations and Future Work

The organization must be equipped with proper internet connectivity and Wi-Fi facilities to effectively monitor the individuals. Passive RFID tags were used for this research. Hence, it cannot be used to calculate the proximity between the users. Since the system was designed to function within an organization, the safety and contact tracing of members outside the organizational premise cannot be guaranteed. The presented work can be extended to monitor elderly people and patients suffering from illnesses such as Alzheimer’s and dementia. Furthermore, active RFID tags can be used to sense the proximity between the users, and hence, the social distancing between the users can be verified. The source code of the proposed implementation is publicly available on the GitHub repository (https://github.com/vravidvk/IoTwristband, accessed on 25 September 2022).

## Figures and Tables

**Table 2:** Comparison of our proposed work with the existing literature.

Feature	Existing Work	Proposed Work
Contact tracing process	Ref. [24] proposed a model that has a wearable band integrated with an RFID receiver, passive RFID tag, and temperature sensor. For the contact tracing process, only the coordinates at which the reader detects a tag were recorded. Thus, contact tracing is dependent on a set of discontinuous GPS coordinates at random locations.	Our work continuously records and stores both the scanned location coordinates of the users and the live GPS location coordinates of the users in the online repository. Thus, we derive an accurate path that can be visualized in detail for contact tracing.
Geofencing	Ref. [23] proposed a geofencing approach dependent on a secondary device such as a mobile phone. The wearable band in this case pairs with the mobile through Bluetooth, and if the band is not in proximity of the phone, it is assumed that the subject is away from the geofenced area. The ease of use is not guaranteed, as the wearable device and the mobile app have to be properly calibrated by following a set of steps.	In our work, the prototype does not depend on another device to check for geofencing. Instead, the geofencing happens in the background all the time via the location coordinates collected by the GPS module and by the coordinates collected during the issue of the wristband. Hence, as soon as the user breaches the geofenced location, an alert is issued.
Status of the user	Ref. [37] presented a social interaction tracking system based on Bluetooth Low Energy (BLE). It uses social interaction-based infection prediction methods to try to overcome the problem of tracking and properly retrieving patients’ past interactions. A mobile app collects information about other nearby phones and uploads it to a cloud storage service with a timestamp and optional GPS position. They devised algorithms to calculate the risk levels associated with each of the patient’s encounters and forecast the likelihood of infection.	Our system is not only effective in ensuring adherence to COVID-19 protocols, but it also takes into account the health status of each user. We do not depend on vaccination certificates, old data, or secondary devices for maintaining workplace safety; instead, the current health status of the users is constantly updated upon receiving new test results each time.
Database management and analysis	Ref. [19] does not inform the members belonging to an organization about the number of sick, healthy, and recovered people present within the organizational premise. The paper only presents a partial idea about the data stored in the database.	Our work maintains an effective database about the details of the users, which can be accessed and viewed by the organization as desired. It also sends the users a daily analysis, compiled over time, of the number of quarantined, recovered, and healthy members present within the organization.

## Data Availability

The datasets generated or analysed during the study are available from the corresponding author on reasonable request.

## Appendix A. Circuit Diagrams and Results

Figure A1 represents the circuit diagram of the wristband components and Figure A2 displays the circuit diagram of the RFID reader.
